# 

**Published:** 1906-06-30

**Authors:** 


### CONTENTS.

**Complete Hospital Co-operation** - 221
**Spectacles through Charity for School-Children** 222
**Annotations** - 223
**Medical Opinion and Movement** - 224
**Hospital Clinics—**
A Clinical Lecture on the Disinfection and Care of the Hands - 225
**Practical Notes on Diagnosis and Treatment** - 227
**The General Practitioner's Column—**
Ringworm and its Cure - 228
Two Cases of Grave Complications of Purulent Ear Disease - 229
**Remedies and their Uses** - 230
**Special Analytical Health Commission on Tinned and Preserved Food** - 231
**New Appliances and Things Medical** - 232
**The Book World of Medicine and Science** - 233
**Medical Appointments and Vacancies** - 233
**Hospital Administration—**
Current Hospital Topics - 234
Harrogate and Knaresborough Isolation Hospital - 235
Hospital Meetings - 237
**To-day and To-morrow** - 238
**Social and Poor Law Problems** - 239
**Editor's Letter-Box** - 240

*NURSING SECTION.*

**Notes on News from the Nursing World—**
Lady Minto and English Nurses for India—Queen Victoria's Jubilee Institute—Nursing in Volunteer Medical Hospitals—Probationers in their Teens—The New Maternity Department at the London Hospital—Nursing at a Modern Poor-law Infirmary—A Theatre Sister for Grimsby—Enteric Patients without Nurses—Five Nurses in One Bedroom—A Scandal at East Ham—Imperial Military Nursing Service—Newcastle Nurses and the Pension Fund—The Victims of the Highgate Tram Accident—Increase of the Staff at Kingston Infirmary—A Badge for Midwives—Short Items - 183-186
**The Nursing Outlook** - 187
**Abdominal Surgery** - 188
**The Nurse's Clinic** - 189
**Incidents in a Nurse's Life** - 190
**The Evolution of a Poor-Law Training School** - 191
**Moving a Patient Suffering from Heart Disease and Dropsy** - 193
**The Management of Children** - 193
**Grand Bazaar at the Albert Hall in Aid of Great Northern Central Hospital** - 194
**Everybody's Opinion** - 194
**Appointments** - 195
**Presentations** - 195
**Death in Our Ranks** - 195
**Novelties for Nurses** - 195
**Notes and Queries** - 196
**For Reading to the Sick** - 196

